# Prolonged hypoglycemia after the resection of pheochromocytoma in a hemodialysis patient

**DOI:** 10.1002/iju5.12770

**Published:** 2024-08-22

**Authors:** Yuya Maezawa, Masaki Kobayashi, Masanori Murakami, Ayumi Yamamura, Shotaro Naito, Hajime Tanaka, Soichiro Yoshida, Tetsuya Yamada, Yasuhisa Fujii

**Affiliations:** ^1^ Department of Urology Tokyo Medical and Dental University Tokyo Japan; ^2^ Department of Molecular Endocrinology and Metabolism Tokyo Medical and Dental University Tokyo Japan; ^3^ Department of Nephrology Tokyo Medical and Dental University Tokyo Japan

**Keywords:** hemodialysis, hypoglycemia, pheochromocytoma

## Abstract

**Introduction:**

Hypoglycemia occasionally develops after the resection of pheochromocytoma due to decreased catecholamine secretion. The unique glucose metabolism in dialysis patients may potentially affect postoperative hypoglycemia, although few reports have focused on this issue.

**Case presentation:**

A 47‐year‐old woman who had chronic renal failure under hemodialysis was diagnosed with right pheochromocytoma and underwent an adrenalectomy. Three hours after surgery, she experienced symptomatic hypoglycemia, which was repeated until postoperative day 3. Continuous glucose infusion was required until postoperative day 8 and asymptomatic hypoglycemia was repeated until postoperative day 11.

**Conclusion:**

In hemodialysis patients, more careful management of hypoglycemia may be required after the resection of pheochromocytoma compared with non‐hemodialysis patients.

**Keynote message**

We documented a case of a hemodialysis patient with right pheochromocytoma who suffered from prolonged hypoglycemia following adrenalectomy. We propose that some mechanisms unique to end‐stage renal disease contributed to persistent postoperative hypoglycemia and conclude that more careful management of hypoglycemia after the resection of pheochromocytoma may be necessary for hemodialysis patients compared with non‐hemodialysis patients.

**Abbreviations & Acronyms**
CT: computed tomography
MIBG: metaiodobenzylguanidine
MRI: magnetic resonance imaging
POD: postoperative day

## Background

The incidence of pheochromocytoma is rare, reported to be 8 per 1 000 000 person‐years annually.^1^ Pheochromocytoma generally affects glucose metabolism by secreting catecholamines, surgical tumor resection is standard practice, and postoperative hypoglycemia occasionally develops due to a reduction in catecholamines levels after the surgery.^2^, ^3^ Therefore, following the resection of pheochromocytoma, close attention to postoperative hypoglycemia and a change in circulatory dynamics is required. Some case studies have focused on managing circulatory dynamics in hemodialysis patients with pheochromocytoma. However, there have been few reports emphasizing the association between end‐stage renal disease and postoperative hypoglycemia, probably due to the rarity of this comorbid condition.^4^, ^5^

Generally, postoperative hypoglycemia after the resection of pheochromocytoma is usually temporary and rarely prolonged.^6^, ^7^ However, we experienced the case of a hemodialysis patient in whom symptomatic hypoglycemia was repeated for 3 days after the resection of pheochromocytoma. In this case report, we highlight the association between end‐stage renal disease and prolonged hypoglycemia after the resection of pheochromocytoma.

## Case presentation

**Fig. 1:**
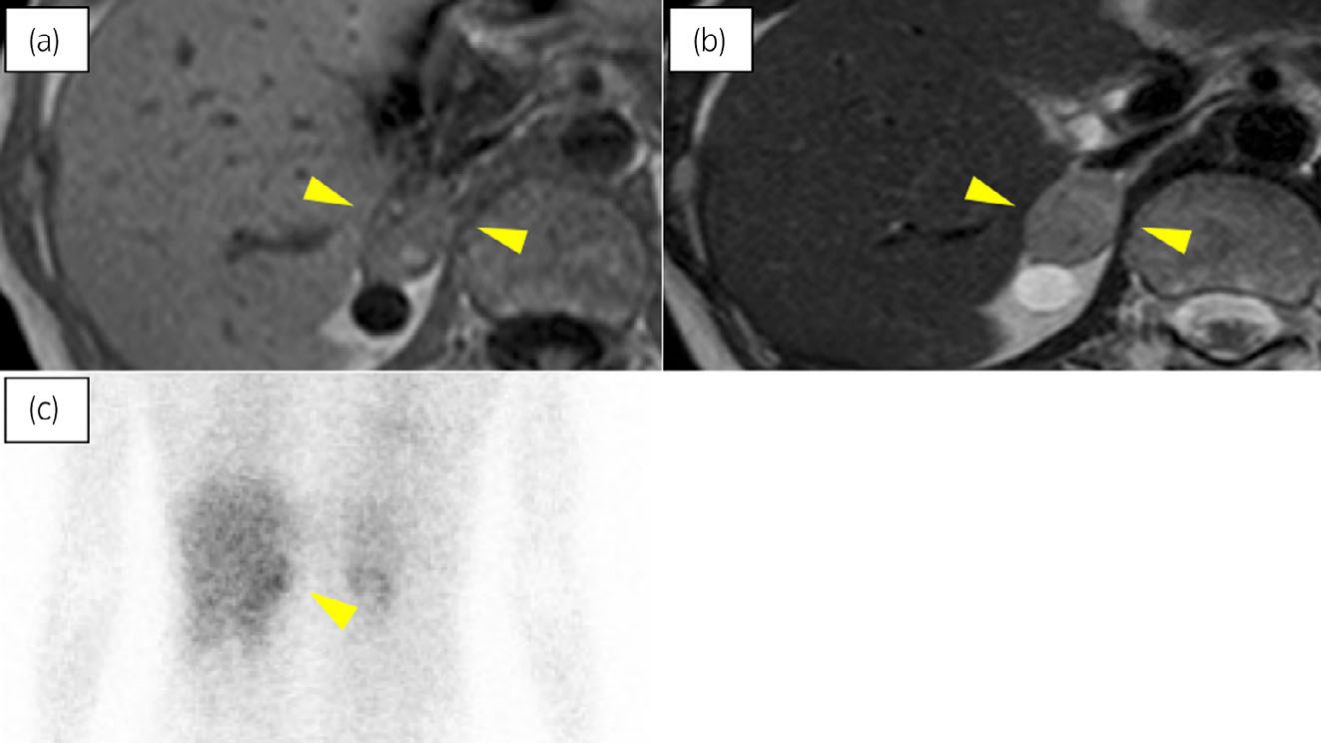
Images of the pheochromocytoma. (a) The right adrenal tumor on T1‐weighted MRI, (b) the right adrenal tumor on T2‐weighted MRI, (c) ^123^I‐MIBG scintigraphy showing high accumulation in the right adrenal gland.

The patient was a 47‐year‐old woman. She had received hemodialysis for the past 10 years because of chronic renal failure that developed from acute kidney injury associated with bone marrow transplantation for aplastic anemia. She had no additional medical history or family history related to pheochromocytoma and had a good performance status. A right adrenal tumor of 11 mm in size was indicated incidentally on abdominal ultrasonography by a previous doctor. Two years later, her blood pressure had increased, and CT and MRI scans revealed that the right adrenal tumor size had increased up to 23 mm (Fig. 1a,b). Therefore, she was referred to our hospital for further examination.

Laboratory tests revealed high levels of serum catecholamines as follows: adrenaline 0.08 ng/mL (normal range: 0–0.17 ng/mL), noradrenaline 1.7 ng/mL (normal range: 0.15–0.57 ng/mL), dopamine 0.22 ng/mL (normal range: 0–0.03 ng/mL), metanephrine 200 pg/mL (normal range: 0–130 pg/mL) and normetanephrine 948 pg/mL (normal range: 0–506 pg/mL). A 24‐h urine catecholamine test was not conducted due to anuria. ^123^I‐MIBG scintigraphy showed an accumulation on the right adrenal tumor (Fig. 1c). Based on these findings, the patient was diagnosed with pheochromocytoma. Her preoperative fasting serum glucose level and HbA1c result were 86 mg/dL and 5.0%, respectively. Her blood pressure was controlled well with doxazosin mesylate 17 mg treatment.

Hemodialysis was continued perioperatively three times a week. The dry weight was set to be higher than usual by 1.0 kg to increase circulating plasma volume and prevent perioperative hypotension. Open right adrenalectomy was performed, operation time was 3 h 21 min, and her blood loss was 120 mL. Her systolic blood pressure increased temporarily to 178 mmHg during manipulation near the tumor. Otherwise, circulatory dynamics was controlled well by phentolamine mesylate, nicardipine hydrochloride, and noradrenaline administration intraoperatively. A total of 5 units of insulin were administered during the surgery and intraoperative serum glucose level ranged from 114 to 240 mg/dL. After surgery, she was transferred to the intensive care unit and monitored carefully. The pathological diagnosis was pheochromocytoma.

**Fig. 2:**
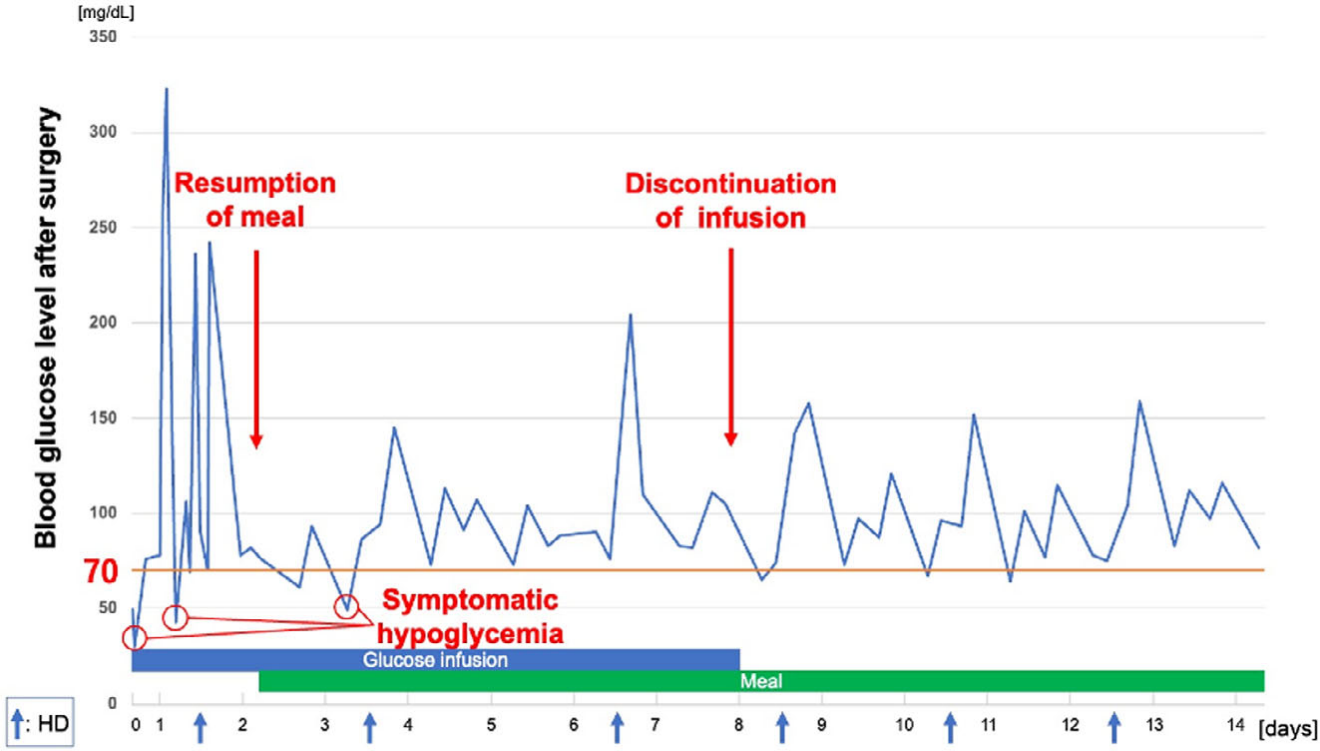
Trend of blood glucose level and timing of symptomatic hypoglycemia after surgery. The monitoring consists of blood measurement until POD 2 and capillary measurement after POD 2.

After surgery, her serum glucose level was monitored every 3 h under administration of 5% glucose at a rate of 20 mL/h. Three hours after surgery, she presented with sweating and, at this time, her serum glucose level was 49 mg/dL. The glucose concentration of the infusion was changed from 5% to 10%. Nevertheless, symptomatic hypoglycemia (serum glucose levels <70 mg/dL) that required a bolus of glucose was observed on POD 1. Even after starting a meal on POD 2, symptomatic hypoglycemia was repeated until POD 3 (Fig. 2). After POD 4, her blood glucose level stayed at more than 70 mg/dL and continuous infusion of glucose was stopped on POD 8. On the morning of POD 5, blood glucose level was 73 mg/dL and serum insulin concentration was 19.1 μU/mL (normal range: 1.7–10.4 μU/mL). After discontinuing glucose infusion, asymptomatic hypoglycemia recurred several times until POD 11, although her blood glucose level stabilized gradually. After POD 12, there was no episode of hypoglycemia, and she was discharged on POD 15.

## Discussion

We reported a hemodialysis patient who had suffered from prolonged hypoglycemia after the resection of pheochromocytoma. To our knowledge, this is the first case report focusing on postoperative hypoglycemia in a hemodialysis patient with pheochromocytoma.

Generally, hypoglycemia after the resection of pheochromocytoma is caused by a reduction in catecholamine levels.^2^ Pheochromocytoma often causes hyperglycemia by secreting catecholamines, which induce gluconeogenesis in the liver and inhibit pancreatic insulin secretion through the alpha‐2 adrenergic receptor. Catecholamines also enhance peripheral insulin resistance through beta‐adrenergic receptors. The decrease in catecholamines after pheochromocytoma removal induces a decline in gluconeogenesis and a relative increase in insulin secretion, resulting in hypoglycemia.^2^ In addition, end‐stage renal disease harbors decreased insulin clearance and impaired gluconeogenesis in the kidney, leading to an imbalance between insulin and glucose levels, as observed in this case on POD 5.^8^ Considering these mechanisms, hypoglycemia following the resection of pheochromocytoma in a hemodialysis patient may be more likely to occur and be prolonged.

In this case, insufficient glucose administration may have also contributed to prolonged hypoglycemia. This patient presented with anuria, which required low‐volume peripheral infusion including glucose to prevent overhydration, even though symptomatic hypoglycemia was prolonged. Administration of a higher concentration glucose solution via a central venous catheter could have provided better blood glucose control. In addition to 11 mg of phentolamine mesylate, a total of 5 units of insulin was intraoperatively used to prevent hyperglycemia, but the amount was not enough to affect postoperative blood glucose level. At the very least, it is difficult to link the insulin and phentolamine mesylate administered during the surgery to the persistence of hypoglycemia. Therefore, it is reasonable to assume that end‐stage renal disease was mainly related to prolonged hypoglycemia.

In this case, postoperative hypoglycemia was significantly prolonged despite continuous glucose administration, possibly due to the specific conditions related to end‐stage renal disease. After the resection of pheochromocytoma in hemodialysis patients, blood glucose levels should be monitored strictly for a longer duration and preparations should be made for persistent postoperative hypoglycemia compared with non‐hemodialysis patients.

## Author contributions

Yuya Maezawa: Writing – original draft. Masaki Kobayashi: Writing – original draft; writing – review and editing. Masanori Murakami: Writing – review and editing. Ayumi Yamamura: Writing – review and editing. Shotaro Naito: Writing – review and editing. Hajime Tanaka: Writing – review and editing. Soichiro Yoshida: Writing – review and editing. Tetsuya Yamada: Writing – review and editing. Yasuhisa Fujii: Supervision; writing – review and editing.

## Conflict of interest

The authors declare no conflict of interest.

## Approval of the research protocol by an Institutional Reviewer Board

Not applicable.

## Informed consent

Written informed consent was obtained from the patient.

## Registry and the Registration No. of the study/trial

Not applicable.
